# Clusterin/Apolipoprotein J immunoreactivity is associated with white matter damage in cerebral small vessel diseases

**DOI:** 10.1111/nan.12248

**Published:** 2015-06-17

**Authors:** Lucinda Craggs, Julie Taylor, Janet Y. Slade, Aiqing Chen, Christian Hagel, Gregor Kuhlenbaeumer, Anne Borjesson‐Hanson, Matti Viitanen, Hannu Kalimo, Vincent Deramecourt, Arthur E. Oakley, Raj N. Kalaria

**Affiliations:** ^1^ Neurovascular Research GroupInstitute for Ageing & HealthNewcastle UniversityNewcastle Upon TyneUK; ^2^ Institute of NeuropathologyUniversitätsklinikum Hamburg‐EppendorfHamburgGermany; ^3^ Department of Molecular NeurobiologyInstitute of Experimental MedicineUniversity of KielKielGermany; ^4^ Institute of Neuroscience and PhysiologySalhgrenska Academy at Göteborg UniversityGoteborgSweden; ^5^ Department of Clinical NeurosciencesKarolinska InstituteHuddinge HospitalStockholmSweden; ^6^ Department of NeuropathologyHelsinki UniversityHelsinkiFinland; ^7^ Univ Lille Nord de FranceExcellence Laboratory DISTALZEA1046Histology and Pathology DepartmentLille University HospitalLilleFrance

**Keywords:** Alzheimer's disease, CADASIL, cerebral amyloid angiopathy, cerebrovascular disease, clusterin, granular osmiophilic material

## Abstract

**Aim:**

Brain clusterin is known to be associated with the amyloid‐β deposits in Alzheimer's disease (AD). We assessed the distribution of clusterin immunoreactivity in cerebrovascular disorders, particularly focusing on white matter changes in small vessel diseases.

**Methods:**

Post‐mortem brain tissues from the frontal or temporal lobes of a total of 70 subjects with various disorders including cerebral autosomal dominant arteriopathy with subcortical infarcts and leukoencephalopathy (CADASIL), cerebral amyloid angiopathy (CAA) and AD were examined using immunohistochemistry and immunofluorescence. We further used immunogold electron microscopy to study clusterin immunoreactivity in extracellular deposits in CADASIL.

**Results:**

Immunostaining with clusterin antibodies revealed strong localization in arterioles and capillaries, besides cortical neurones. We found that clusterin immunostaining was significantly increased in the frontal white matter of CADASIL and pontine autosomal dominant microangiopathy and leukoencephalopathy subjects. In addition, clusterin immunostaining correlated with white matter pathology severity scores. Immunostaining in axons ranged from fine punctate deposits in single axons to larger confluent areas with numerous swollen axon bulbs, similar to that observed with known axon damage markers such as non‐phosphorylated neurofilament H and the amyloid precursor protein. Immunofluorescence and immunogold electron microscopy experiments showed that whereas clusterin immunoreactivity was closely associated with vascular amyloid‐β in CAA, it was lacking within the granular osmiophilic material immunolabelled by NOTCH3 extracelluar domain aggregates found in CADASIL.

**Conclusions:**

Our results suggest a wider role for clusterin associated with white matter damage in addition to its ability to chaperone proteins for clearance via the perivascular drainage pathways in several disease states.

## Introduction

Clusterin, also known, as apolipoprotein J, is a highly conserved 78–80 kDa heterodimeric glycoprotein expressed in a wide variety of tissues and cells, encoded by a single copy gene on chromosome 8 in man [1]. This gene encodes a 449 amino acid precursor polypeptide which is cleaved to form the mature secreted clusterin protein; a heterodimeric glycoprotein consisting of two disulphide linked α‐ and β subunits which functions to scavenge and chaperone damaged proteins in the extracellular compartment of tissues [1, 2]. Other functional variants of clusterin are generated by alternative splicing of the mRNA, producing a cytoplasmic variant, which can translocate to the nucleus and influence cell survival [1, 3]. The apparent ubiquitous presence of clusterin has the potential for it to be used as a biomarker for health and in disease.

Genome‐wide association studies have identified clusterin as one of the risk genes associated with Alzheimer's disease (AD) [4, 5]. Previous studies have reported a correlation between raised clusterin levels in plasma with rapid clinical progression and disease severity of AD [6, 7], although this association is not consistently confirmed [8, 9]. Studies measuring protein levels in cerebrospinal fluid (CSF) revealed elevated CSF clusterin in AD cases compared with controls [10], and a study of non‐demented and mild cognitive impairment patients revealed increased CSF clusterin was related to amyloid β‐associated brain atrophy [11].

Further to the role in cerebral amyloid angiopathy (CAA) and AD, clusterin may be involved in pathological changes in cerebral autosomal dominant arteriopathy with subcortical infarcts and leukoencephalopathy (CADASIL), the most common type of hereditary small vessel disease (SVD), caused by mutations in the *NOTCH3* gene [12, 13]. CADASIL is pathologically distinguished by Periodic Acid Schiff‐positive cerebral vessels and the presence of deposits, 0.2–2 μm in size, of granular osmiophilic material (GOM) within vessel walls [14, 15, 16]. Our previous work demonstrated that NOTCH3 extracellular domain (N3ECD) accumulates in GOM and that N3ECD‐positive GOM deposits are found in high abundance around blood vessels of the grey and white matter (WM), and around pial vessels and the meninges [16, 17]. Recent studies showed that the extracellular domain of mutant NOTCH3 within GOM deposits may attract other proteins in a ‘snowball effect’ [12], and a few intriguing proteins enriched within the vessel walls have been identified in CADASIL [12, 13]. Both the latter studies had indicated that clusterin immunoreactivity was associated with GOM deposits within the brain microvasculature in CADASIL subjects.

Here, we investigated clusterin protein expression in post‐mortem brain tissue from different dementing disorders including CADASIL, CAA and hereditary stroke disorders. We used different immunocytochemical methods to examine patterns and quantify clusterin immunostaining in the frontal lobe with particular focus on cerebral vessels and WM pathology.

## Methods

### Subjects

Post‐mortem formalin‐fixed paraffin‐embedded brain tissue was obtained from a number of sources. While the majority of the total of 70 cases were from the Newcastle Brain Tissue Resource (NBTR), and the rest were obtained from the MRC London Brain Bank for Neurodegenerative Diseases, the MRC Sudden Death Brain and Tissue Bank, University of Edinburgh, Neurology Department, Ludwig Maximilians University, Germany, Department of Neuropathology, University of Hamburg, Germany, and Department of Neuropathology, Upsalla University, Sweden. The cohort included three cognitively normal control groups and seven different dementing disorders [16, 18] including SVD, CADASIL, hereditary multi‐infarct dementia of Swedish type (Swedish hMID), pontine autosomal dominant microangiopathy and leukoencephalopathy (PADMAL), CAA, dementia with Lewy bodies (DLB), and AD (Table 1).

**Table 1:** Demographics of subjects in the cohort to analyse clusterin immunostaining in vascular and neurodegenerative disorders

	Young controls	Old controls	95+ Controls	SVD	CADASIL	Swedish hMID	PADMAL	DLB	CAA	AD
*N* (total 70)	8	8	5	8	10	4	5	7	7	8
Mean age (years)	57.6	85.8	99.4	83.4	58.5	42.5	50.0	75.4	83.3	76.8
Range (years)	46–65	78–94	95–104	68–96	77–68	30–48	42–59	71–85	77–88	63–87
Gender (M/F)	5/3	2/6	1/4	2/6	7/3	2/2	2/3	4/3	3/4	3/5
Mean age at onset				n/a	46.2	33.8	40.8	69.0	74.0	61.5
Duration of disease				n/a	12.6	8.8	9.2	6.6	10.3	8.0
Median WM score	2	2.5	2	2	3	2	3	2	3	2
Range	2–3	2–3	2–2.5	1.5–3	2–3	1–2	2–3	1–3	2–3	2–2.5

Mean age of young controls was not different from the CADASIL and PADMAL subjects. The mean age of the old controls was not different from SVD or CAA subjects. Mean age of Swedish hMID group was different from young controls and CADASIL (*P* < 0.05) but not from PADMAL. WM pathology (WM Score) for each case was scored by two people (LJLC an JLT), and the average score was used in the analysis; the median WM score was significantly different between groups (*P* = 0.016), where CADASIL, PADMAL and CAA had highest WM scores of 3, followed by the old controls with median score of 2.5, and the remaining groups had median score of 2; SVD 95+ Controls, young controls, DLB, AD, Swedish hMID (Kruskal–Wallis H test). The post‐mortem interval between death and immersion fixation of tissue ranged from 9 to 96 h. The length of fixation of tissues prior to paraffin embedding ranged from 1 to 7 months.

95+ Controls, controls aged over 95 years; AD, Alzheimer's disease; CAA, cerebral amyloid angiopathy; CADASIL, cerebral autosomal dominant arteriopathy with subcortical infarcts and leukoencephalopathy; DLB, dementia with Lewy bodies; PADMAL, pontine autosomal dominant arteriopathy microangiopathy and leukoencephalopathy; SVD, small vessel disease; Swedish hMID, hereditary multi‐infarct dementia of the Swedish type.

Dementia diagnoses and neuropathological examination were performed essentially as described previously [18]. Briefly, subjects who were part of prospective studies in Newcastle had undergone extensive and comprehensive clinical and cognitive assessments, including the Cambridge Examination for Mental Disorders in the Elderly and Mini‐Mental State Exam. The diagnosis of AD was in accordance with the National Institute of Neurological and Communicative Disorders and Stroke/Alzheimer's Disease. Clinical diagnosis of vascular cognitive impairment (VCI) or VaD was consistent with the proposed Harmonization Guidelines for VCI [19] and VCD [20]. The control groups consisted of subjects with no evidence of dementia as determined by retrospective or prospective assessment at recruitment by the absence of neurological or psychiatric disorder. SVD cases from other centres were retrospectively assessed according to the criteria for VCI or dementia we used. Use of brain tissue was approved by the local research ethics committee 1 of the Newcastle upon Tyne Hospitals NHS Foundation Trust, the NBTR committee and the internal review boards of the Institutional or Departmental brain collections at other sites including Upsalla (Sweden), Hamburg (Germany) and Munich (Germany).

### Immunohistochemistry (IHC)

Ten micrometre thick tissue sections from the frontal lobe, approximate Brodmann area 9 (BA9), were immunohistochemically stained with two different anti‐clusterin antibodies (dilution 1:200–2000, Ab#69644 and Ab104652, Abcam, Cambridge, UK). Ten images from the neocortex and underlying WM were captured using a Zeiss Axioplan 2 (Zeiss, Oberkochen, Germany) microscope and analysed using Image Pro Plus software (V.4.0, Media Cybernetics, Silver Spring, MD, USA). The per cent area of an image covered by the stain was assessed (per cent area; %A) as an indication of the extent of clusterin immunostaining. To assess the intensity of the immunostaining per area (%A) and between sections, we also determined the integrated optical density as a measure of the mean immunoreactivity (IR) for each sample. The Image Pro analysis software assigns an individual IOD between 0 and 255 grey levels for each stained pixel in the delineated object, depending on the stained pixel intensity. All of the individual grey levels are summed for the entire stained object, and this total sum is divided by the number of pixels contained within the object, which gives the mean pixel IOD per object. From these mean IOD's per object, a total IOD was determined for all stained objects within an image or series of images.

Clusterin immunostaining in axons was scored in each case as follows: 0, no axonal staining; 1, infrequent, short punctate staining; 2, infrequent staining of individual axons; 3, patches of punctate staining with swollen axonal lengths; 4, tracts of densely stained and swollen axons but unstained areas still apparent; 5, complete areas of densely stained and swollen axons (Figure S1). Specificities of all antibodies were verified by including irrelevant antibodies or rabbit preimmune serum in incubated sections with antigens of interest. We also used two different clusterin antibodies, which gave similar results. Clusterin antibody specificity (antibody Ab#69644) was confirmed by immunoblotting of frozen human brain tissue extracts (Supplemental methods), where a 38 kDa band representing secreted clusterin isoforms was detected [2]. In one CADASIL subject, a second band at approximately 50 kDa representing the nuclear isoform was detected (Figure S2).

### Immunofluorescence (IF)

Six micrometre thick formalin‐fixed paraffin‐embedded tissue sections were cut from the same frontal lobe blocks as for IHC, and underwent antigen retrieval before blocking with 10% normal horse serum/PBS. Antigen retrieval was performed by immersing sections in boiling 0.01M citrate (pH6) and leaving to rest for 20 min. The sections were then transferred into deionized water. Sections were incubated overnight at 4°C with primary antibodies to clusterin, smooth muscle α‐actin (clone 1A4, 1:500, DAKO Cytomation, Glostrup, Denmark), anti‐amyloid β 1–40 (Ter40; 1:200 [21]), anti‐amyloid β 1–42 (Ter42: 1:200 [21]) and anti‐N3ECD (A1‐1, 1:10,000 [22]). For co‐localization after removal of primary antibodies, the sections were washed in PBS and incubated for 45 min with the following secondary antibodies: goat anti‐mouse IgG conjugated DyLight 488 at 1:200 (product no. 35502; Thermo Scientific, Rockford, IL, USA) and goat anti‐rabbit IgG conjugated DyLight 550 at 1:200 (product no. 84541; Thermo Scientific) or donkey anti‐goat Alexa Fluor 488 (product no. 1084434; Life Technologies, Grand Island, New York, USA) at 1:500. Sections were then washed in PBS before DAPI staining and mounting in Vectashield (Vector Laboratories, Burlingame, CA, USA). Sections were imaged using a Leica TCS SP2 upright confocal microscope and co‐localization graphs (cytoflourograms) of antibodies were produced using Leica Software LCS 2.61 (Leica Microsystems, Wetzlar, Germany). Co‐localization analysis of proteins was performed using the Just Another Co‐localization Plugin (JaCoP) tool in Image J (National Institutes of Health, Bethesda, Maryland, USA) [23].

### WM pathological assessment

Tissue sections from the frontal lobe were stained with luxol fast blue and cresyl fast violet (LFB/CFV) to determine WM pathology. A scoring system based on the methods of Deramecourt *et al*. [24] was implemented as follows from a scale of 0 and 3: 0: normal appearance of WM; 1: slight loosening of WM and/or slightly dilated perivascular space; 2: loosening of WM with bubbly appearance and/or dilated perivascular space; and 3: severe loosening of WM with bubbly appearance and severe pallor, swollen axons visible [24]. The WM pathology for each case was scored by two operators (JT and LC) and the average score used in the analysis.

### Immunogold electron microscopy

Brain tissues from CADASIL patients were sampled from the neocortex and WM of the temporal lobes and the cerebral meninges, and stained using immunogold staining procedures as described previously [16]. The non‐osmicated 700 Å thick epoxy‐resin sections were etched with two changes of 3% sodium meta‐periodate for 20 min before heating in 0.01 mol/l citrate buffer (pH 6) at 90°C for 10 min. After blocking with 5% bovine serum albumin, 5% normal goat serum and 0.1% gelatine in Tris buffered saline, the grids were incubated for 90 min at room temperature with anti‐N3ECD (A1‐1 antibody, 1:4000) or anti‐clusterin (Ab69644, 1:200, Abcam) antibodies in buffer [Tris buffered saline (pH 7.4) containing 1% bovine serum albumin and 0.1% Tween‐20]. The sections were rinsed with three changes of buffer before incubation in EM‐grade goat anti‐rabbit IgG 5‐nm gold probes (1:30; BB International, Madison, WI, USA) for 90 min. Gold particle enhancement was performed using a Silver Enhancing Kit for Light and Electron Microscopy (BB International). Sections were postfixed in 2% buffered glutaraldehyde and contrasted with uranyl acetate and lead citrate. Electron microscopy (EM) images were taken using a Philips 201 transmission electron microscope coupled to a Gatan multiscan camera, model 791 (Gatan, Pleasanton, CA, USA).

### Statistical analysis

Results were analysed using spss (IBM, V 19.0, IBM Corporation, Armonk, NY, USA). Where relevant, following analysis of variance (anova), post hoc analysis was performed using Tukey's post hoc. Axonal damage scores used ordinal data and were analysed for between‐group effects using Kruskal–Wallis H test followed by Mann–Whitney *U* test post hoc. For axonal staining, disease groups were compared the respective age‐matched controls and the differences between the groups tested using Mann–Whitney *U* test. Histograms were presented as mean ± 2 SEM, and values of *P* < 0.05 were considered significant.

## Results

### Clusterin immunolocalization in the neocortex

**Figure 1:**
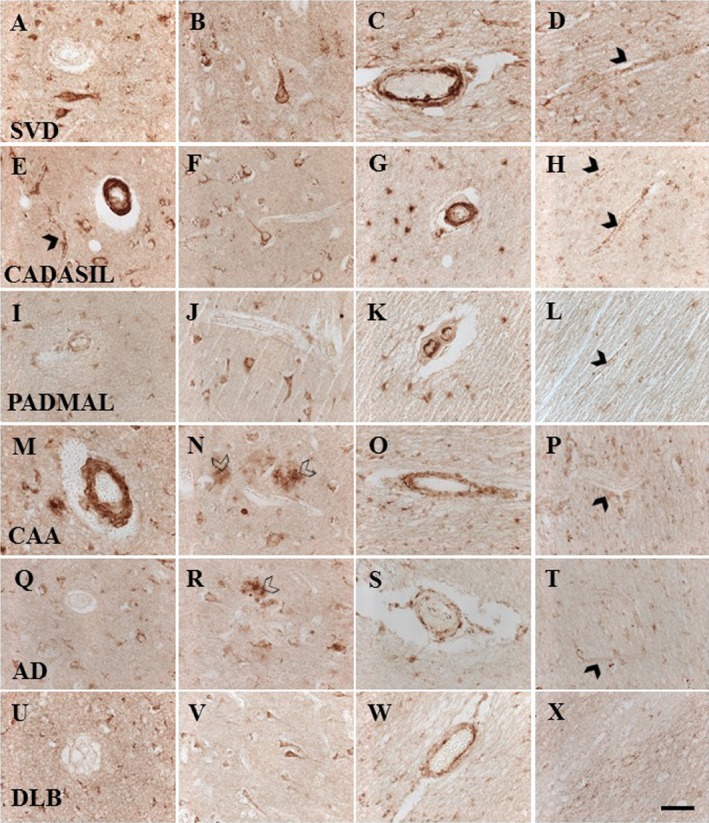
Representative images of clusterin immunostaining in the neocortex, WM and blood vessels in different disorders. Case labels show images for each disorder: **A**–**D**, an 81 year‐old female with SVD; **E**–**H**, a 44 year‐old female with CADASIL; **I**–**L**, a 45 year‐old male with PADMAL; **M**–**P**, an 86 year‐old female with CAA; **Q**–**T**, an 84 year‐old female with AD; **U**–**X**, a 75 year‐old male with DLB. Columns represent immunoreactivity in arterioles of the cortex (**A**, **E**, **I**, **M**, **Q** and **U**), neurones and plaques in the cortex (**B**, **F**, **J**, **N**, **R**, **V**), arterioles in the WM (**C**, **G**, **K**, **O**, **S**, **W**) and capillaries in the WM (**D**, **H**, **L**, **P**, **T**, **X**). Note very distinct arteriolar immunostaining in CADASIL and PADMAL (**G** and **K**). Arrowheads indicate capillaries. Open arrowheads indicate clusterin immunostaining in amyloid plaques. Magnification bar = 50 μm.

**Figure 2:**
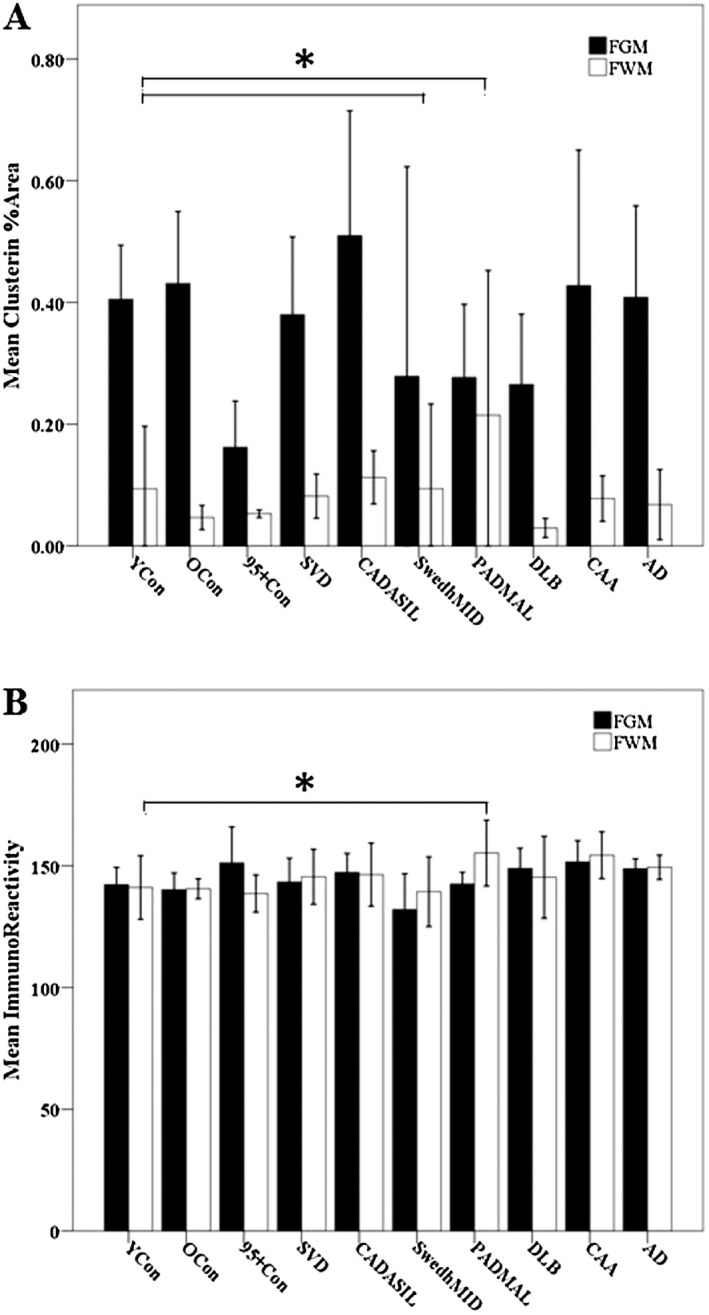
(**A**) Percent area (%A) clusterin immunostaining in frontal grey matter (FGM) and frontal WM (FWM). There was no significant difference in %A immunostaining in the FGM (*P* > 0.05), but CADASIL and PADMAL groups showed significantly greater %A stain in the FWM (Kruskal–Wallis H test, *P* = 0.013). (**B**) Mean clusterin immunoreactivity derived from IOD (see *Methods*) in the FGM and FWM. There was a general lack of differences in intensity of clusterin immunostaining (*P* > 0.05). Error bars are 2 standard errors of the mean.

Both clusterin antibodies showed positive immunostaining in cerebral vessels in different disorders (Figure 1). Some cortical neurones were intensely immunostained in all the disorders (Figure 1). Clusterin immunoreactivity was observed as dense granular deposits associated with cortical arterioles in both CADASIL and CAA subjects (1E and 1M, respectively), and frequently in capillaries of CADASIL subjects, often as perivascular punctate deposits (cf. Figure 1 **E**). Clusterin immunostaining was also found in cortical plaques and perivascular amyloid deposits in CAA and AD cases (Figure 1 **N**,**R**). We further observed occasional clusterin immunopositive granular deposits within walls of pial arteries in SVD, and CAA cases but less frequently in CADASIL and PADMAL. However, upon quantification, we found no significant differences in clusterin percent area stained (%A) in the neocortex (Figure 2 **A**,**B**), which avoided any amyloid plaques or vascular deposits and was representative of only neuronal and glial expression. There was no correlation between neuronal clusterin immunostaining and Braak or CERAD scores in the AD subjects (Spearman's Rho *P* > 0.05, data not shown).

### Clusterin immunolocalization with vascular pathology and axon damage

**Figure 3:**
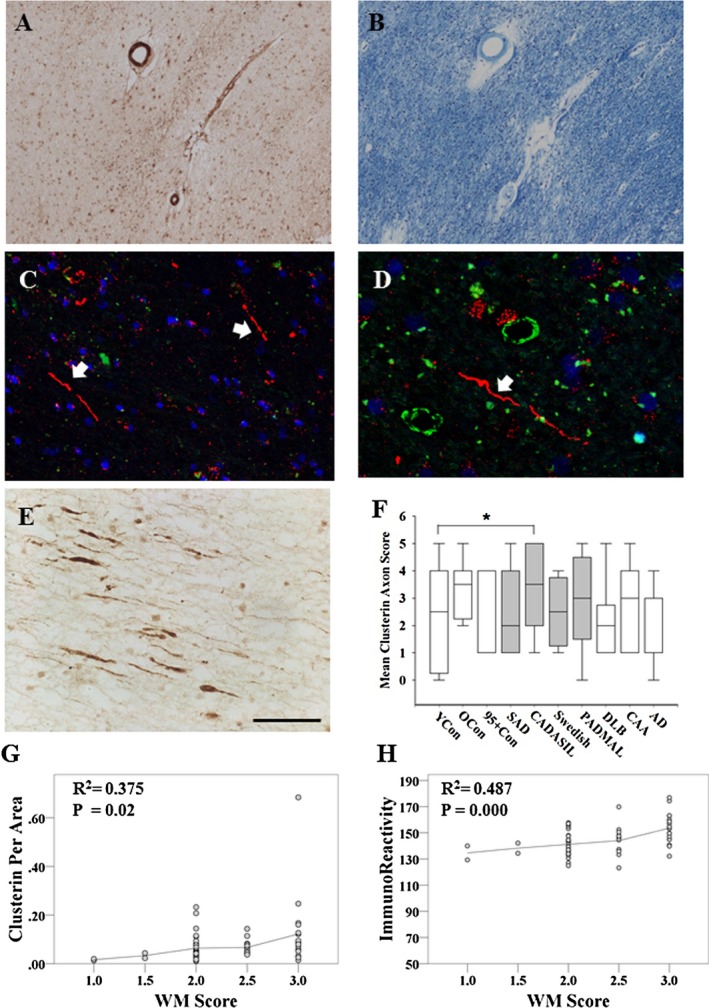
(**A** and **B**) Typical vascular pathology in a 68 year‐old male CADASIL case with p.Arg153Cys *NOTCH* *3* mutation, demonstrating degeneration of arteriole walls and glial reactivity within the white matter (WM); (**A**) clusterin immunostaining; (**B**) LFB/CFV stain. (**C** and **D**) Immunoflourescent labelling of clusterin (arrows) highlights swollen axons in the WM of a 61 year‐old male CADASIL case with p.Arg169Cys mutation (**C**), and a 63 year‐old male AD case (Braak stage VI and CERAD frequent). Colours: clusterin in red, smooth muscle alpha actin in green and DAPI in blue. Note the absence of green in CADASIL case. (**E**) Clusterin‐stained axonal spherules in WM of a 65 year‐old male with CADASIL with p.Arg141Cys *NOTCH* *3* mutation. This case was scored as 5 in axonal clusterin immunostaining scale. (**F**) Quantitative scores of axonal staining in all groups as boxplots. *CADASIL different from young controls (YCon, *P* = 0.040, Mann–Whitney *U* test). (**G**) Scatter graph comparing clusterin percent area (%A) and WM score in all 69 cases, indicating significant positive association between WM scores and larger areas of clusterin immunostaining [percent area (%A)] (Spearman's Rho, *P* = 0.02). (**H**) Scatter graph comparing clusterin IR and WM scores in all 69 cases, where there were significant positive association between WM scores and greater clusterin IR (Spearman's Rho, *P* = 0.000). Error bars in **F** are 2 standard errors of the mean. YCon, Young controls; OCon, old controls; 95+ Con, controls aged over 95 years; SVD, small vessel disease; CADASIL, cerebral autosomal dominant arteriopathy with subcortical infarcts and leukoencephalopathy; Swedish, hereditary multi‐infarct dementia of the Swedish type; PADMAL, pontine autosomal dominant arteriopathy microangiopathy and leukoencephalopathy; DLB, dementia with Lewy bodies; CAA, cerebral amyloid angiopathy; AD, Alzheimer's disease. Magnification bar = 200 μm in **A** and **B**, 70um in **C**, 50um in **D** and **E**.

In the WM, clusterin immunoreactivity was evident in astroglia, in swollen and damaged axons as well as fine granular or punctate deposits in walls of arterioles and along many capillaries (Figure 1 **D**,**H**,**L**,**P**). Using Kruskal–Wallis H tests, we found PADMAL brain tissues to reveal significantly greater %A of clusterin immunostaining in the WM (Figure 2 **A**) compared with the other groups, followed by Swedish hMID, CADASIL and young control groups. Clusterin IR was generally not altered across the different groups (Figure 2 **B**). Upon further examination, clusterin immunostained multiple cell types in the areas of WM damage including glia and blood vessels (Figure 3 **A**,**B**), as well as swollen and damaged axons (Figure 3 **C**–**E**). Axonal immunostaining ranged from fine punctate deposits in single axons to larger confluent areas of axon tracts with many swollen axon bulbs, similar to that observed with known axon damage markers such as non‐phosphorylated neurofilament H (SMI32) and amyloid precursor protein (not shown) [18]. We developed a qualitative scoring scale to assess the differences in the clusterin axon staining in our cases (Figure S1). Kruskal–Wallis H test revealed significant differences in clusterin axon score between the groups (*P* < 0.05), where we found significant increases in clusterin immunostaining within damaged axons in CADASIL compared with young controls despite their similar age (*P* < 0.05, Mann–Whitney *U* test) (Figure 3 **F**). However, there were no differences between young controls and the Swedish hMID and PADMAL groups (*P* = 0.847 and 0.408 respectively, Mann–Whitney *U*). There was also no significant difference between the clusterin axon score of the old controls and SVD, CAA, and AD (*P* = 0.178, *P* = 0.232 and *P* = 0.057 respectively, Mann–Whitney *U*). There was no correlation between the clusterin axon score and age across the control groups (*P* > 0.05, Spearman's Rho).

Median WM scores and the range of WM scores showed the following decreasing order in severity: CADASIL > PADMAL > CAA > old controls > SVD > 95+ controls > young controls > DLB > AD > Swedish hMID (*P* < 0.05, Kruskal–Wallis H test, Table 1). Spearman's rho correlation revealed a relationship between both clusterin %A in the WM and WM vascular pathology scores across the cohort, indicative of a relationship between clusterin and vascular‐type WM damage (Figures 3 **G**,**H**). To investigate clusterin immunostaining in dementias with different primary disease mechanisms (vascular *vs.* neurodegenerative degeneration), cases were grouped into three categories: cognitively normal controls (young controls, old controls and 95+ controls), dementia of vascular origin (CADASIL, SVD, Swedish hMID, PADMAL and CAA) or neurodegenerative origin (AD and DLB). We found there was a trend for a higher median WM score in dementias with a vascular pathology (VasType) (Kruskal–Wallis H test, *P* = 0.059). The %A immunostaining of clusterin compared in this manner showed no significant difference between the groups in neocortex. However, the WM had greater areas of clusterin immunostaining in vascular‐type dementia (Kruskal–Wallis H test, *P* = 0.02), indicating an increase in clusterin expression in the presence of vascular‐associated axon pathology and not in cases with neurodegenerative pathologies where WM was less affected (data not shown).

### Localization of clusterin in CAA and N3ECD deposits

**Figure 4:**
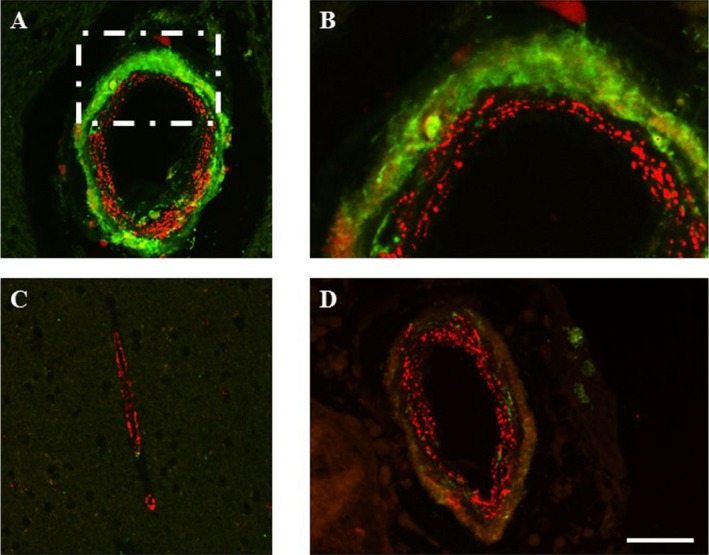
Clusterin and N3ECD immunoreactvities in blood vessels in CADASIL. (**A**) An image of arteriole from a 44 year‐old female CADASIL subject with p.Arg153Cys *NOTCH* *3* mutation, showing clusterin (green) immunostaining is associated within the adventitial layers of the blood vessel wall, while N3ECD positive (GOM) deposits (red) are mostly in the tunica media. (**B**) Higher power image of box in **A**. There was no substantial overlap. (**C**) N3ECD positive deposits or GOM (red) found aggregated within capillary walls, which were negative for clusterin (green); same CADASIL subject as in **A** and **B**. (**D**) N3ECD positive deposits or GOM (red) in pial arteries and meninges but little clusterin (green) immunostaining. Image **D** from a 61 year‐old male CADASIL subject with p.Arg169Cys mutation. Scale bar represents 40 μm in **A**, 20 μm in **B**, 60 μm in **C** and 30 μm in **D**.

**Figure 5:**
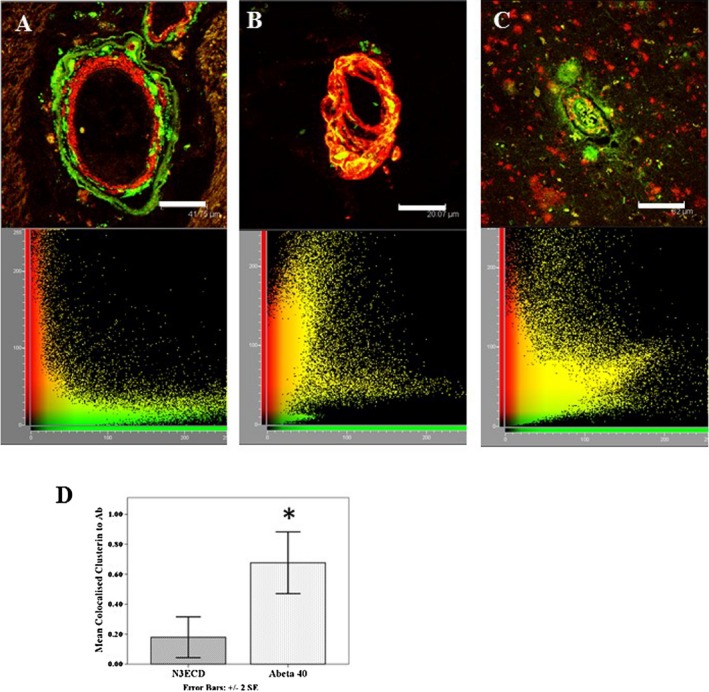
Co‐localization of immunofluorescent labelling of clusterin and aggregated proteins. (**A**) Clusterin (green) accumulates within adventitial layers of arterioles in WM, but was not co‐localized with N3ECD‐labelled GOM (red) in a CADASIL subject with p.Arg153Cys *NOTCH* *3* mutation. Below **A**, cytofluorogram of co‐localization between clusterin (green, *x*‐axis) and N3ECD (red, *y*‐axis), Manders' coefficient for fraction of clusterin overlapping with N3ECD was *M* = 0.017, indicating lack of overlap. (**B**) Clusterin (green) found to accumulate in vessel walls of arterioles with amyloid β 1–40 (red) in the cortex of an 88 year‐old male with cerebral amyloid angiopathy (CAA). Below **B**, cytofluorogram of clusterin (green, *x*‐axis) and amyloid β 1–40 (red, *y*‐axis) with Manders' coefficient for fraction of clusterin overlapping amyloid β 1–40 *M* = 0.999, indicating high overlap. (**C**) Clusterin (green) observed as aggregates in plaques and perivascular amyloid deposits. Amyloid β 1–42 was deposited in further deposits, which were negative for clusterin. Same CAA case as in **B**. Below **C**, cytofluorogram of co‐localization between clusterin (green, *x*‐axis) and amyloid β 1–42 (red, *y*‐axis), Manders' coefficient for fraction of clusterin overlapping amyloid β 1–42 *M* = 0.59. (**D**) Mean Manders' coefficients for the ratio of clusterin to amyloid β 1–40 (Abeta 40) in a CAA case was significantly higher for amyloid β 1–40 compared with clusterin association with N3ECD in a CADASIL case (Mann–Whitney *U*, *P* = 0.024). Magnification bar = 35 μm in **A**, 20 μm in **B** and 82 μm in **C**.

We used immunofluorescent (IF) labelling to compare clusterin immunostaining in N3ECD GOM deposits in CADASIL and amyloid deposits in CAA subjects. IF labelling revealed clusterin immunostaining in ring‐like deposits around vessel walls. These appeared to be larger deposits than N3ECD deposits, and were localized more in the adventitial layers rather than tunica media where N3ECD deposits were observed (Figure 4 **A** and inset, **B**). Although we observed positive N3ECD‐stained deposits within capillary walls (Figure 4 **C**), clusterin immunostaining was relatively less frequently associated with capillaries. There was less consistent immunostaining for clusterin in pial blood vessels or the meninges, despite abundance of N3ECD labelled GOM in pial arteries (Figure 4 **D**). In contrast, clusterin was juxtaposed and co‐localized with vascular amyloid‐β 1–40 but rarely amyloid‐β 1–42 deposits in CAA (Figure S2). We performed quantitative analysis of the co‐localization of clusterin using image analysis plug‐in for Image J (JaCoP [23]). Co‐localization analysis in numerous CADASIL arterioles revealed that clusterin and N3ECD immunoreactivities were localized completely independently of each other (Figure 5 **A**), where the Manders' coefficient for fraction of clusterin overlapping N3ECD was *M* = 0.017 (*P* < 0.001), indicating low level of co‐localization. On the contrary, we found clusterin co‐localized strongly with amyloid‐β 1–40 in CAA, where the Manders' coefficient for fraction of clusterin overlapping amyloid‐β 1–40 was *M* = 0.999 (Figure 5 **B**, *P* < 0.001). Clusterin immunostaining was less strongly associated with amyloid‐β 1–42, where many amyloid‐β 1–42 deposits were negative for clusterin immunoreactivity (Figure 5 **C**). The Manders' coefficient for the fraction of clusterin overlapping with amyloid‐β 1–42 was *M* = 0.59 (*P* < 0.001). The mean Manders' coefficients for the ratio of clusterin to amyloid‐β 1–40 (Abeta 40) in CAA arterioles were significantly higher than Mander's coefficients for the ratio of clusterin association with N3ECD in CADASIL arterioles (Figure 5 **D**, Mann–Whitney *U*, *P* = 0.024).

### Ultrastructural localization and distribution of GOM

**Figure 6:**
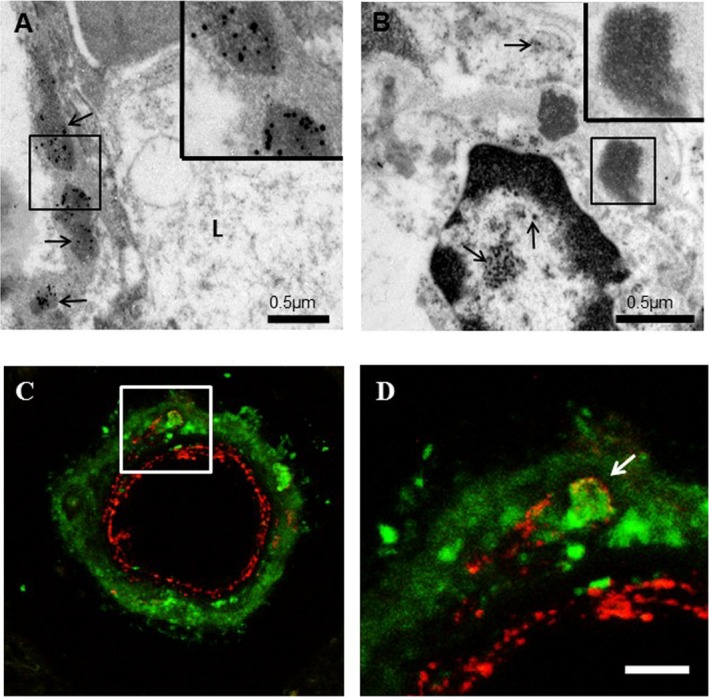
Immunogold labelling in cerebral vessels by electron microscopy in a 68 year‐old male CADASIL patient with p.Arg153Cys mutation. (**A**) 5 nm gold particles representing N3ECD immunoreactivity in a white matter arteriole observed only within GOM deposits in the tunica media (arrows, outlined in box and inset). (**B**) Immunogold labelling with clusterin antibody revealed positive immunoreactivity (arrows) in the nucleus, cytoplasm and extracellular compartment of a vascular smooth muscle cell of an arteriole in the white matter of the same case as **A**. The GOM (outlined in box and inset) shows lack of positivity for clusterin. (**C**) Immunofluorescence with confocal microscopy demonstrating distribution of clusterin (green) and N3ECD (red) antibodies within the wall of a white matter arteriole from the CADASIL patient with p.Arg153Cys mutation. Clusterin antibody stained diffusely within the outer layers of the vessel wall, as well as staining within the cytoplasm of cells within the vessel wall. Some positive N3ECD immunoreactivity was observed in close proximity to a cell with intracellular clusterin immunoreactivity (outlined in box). (**D**) Higher magnification of boxed area in **C**. N3ECD immunoreactivity (red) labelling granular deposits (arrows) surrounding a cell with intracellular clusterin immunoreactivity (green). L, lumen. Magnification bars = (**A** and **B**) 0.5 μm, (inset **A** and **B**) 1 μm, (**C**) 22 μm and (**D**) 7.5 μm.

To confirm the location of clusterin immunostaining within CADASIL vessel walls, we performed immunogold EM with antibodies to N3ECD and clusterin [16]. The N3ECD (A1‐1 antibody) demonstrated strong immunoreactivity within GOM deposits of 0.2–2 μm diameter (Figure 6 **A**). Clusters of gold particles were only found on GOM deposits associated with plasma membranes of vascular smooth muscle cells and pericytes (Figure 6). In contrast, we observed immunoreactivity for clusterin within the cytoplasm of cells and some immunostaining within the nucleus of vascular smooth muscle cells, but there was no evidence of similar positivity for clusterin within GOM deposits (Figure 6 **B**). This was in agreement with observations with the high‐resolution confocal microscopy imaging of sections from CADASIL cases (above). In addition to diffuse clusterin immunostaining within the adventitial layers of arterioles, some adventitial cells clearly demonstrated intracellular clusterin immunostaining (Figure 6 **C**,**D**). As expected, N3ECD stained small punctate deposits within the medial layers of the vessel wall, yet there was clearly some N3ECD punctate immunoreactivity around a cell with intracellular clusterin immunostaining, suggesting this cell was an atrophic vascular smooth muscle cell. Intracellular clusterin immunostaining of vascular cells was not only observed in CADASIL subjects but was evident in other disorders (data not shown).

## Discussion

Our study showed distinct clusterin immunoreactivity in cerebral blood vessels in subjects with different microvascular disorders, including familial early‐onset stroke disorders. Increased clusterin immunoreactivity in the WM of the frontal lobe was most notable. The expression of clusterin in the WM was particularly concomitant with disorders with a vascular component including CAA and PADMAL, a disease we have previously reported to show severe vascular pathology in the frontal lobe [18]. Importantly, clusterin immunostaining in our young control group was of a similar level to some of our disease groups, and in the case of axonal clusterin immunoreactivity, our young control group ranked above DLB, AD and Swedish hMID groups. We have previously reported on the extent of vascular damage in the brains of these cases, where we reported >20% of blood vessels in the FWM of young control cases had arteriolosclerosis equivalent to a disease state [18]. We believe our cohort of cognitively normal young controls may exhibit some levels hypoxic damage owing to their comorbidities (lung cancer cardiovascular disease [18]). These observations are also in general agreement with previous studies [25, 26, 27, 28, 29, 30], which reported clusterin activation subsequent to different types of brain injury, specifically the localization in glial cells within the WM as well as regions of damaged axons. Increased clusterin immunoreactivity was also related to WM damage in aged rats [31], and reported to be activated in astrocytes following middle cerebral artery occlusion in rats [30]. The lack of significant changes in neuronal clusterin expression within the neocortex is consistent with several studies [26, 32, 33]. Our immunohistochemical image analysis did not include clusterin immunoreactivity associated with amyloid plaques and perivascular amyloid in AD and CAA.

Strong clusterin immunoreactivity was evident in both CAA and AD cases, in which clusterin has been previously shown to be associated with amyloid plaques and perivascular amyloid deposits [12, 13, 33, 34, 35]. We also confirm the localization of clusterin with amyloid‐β 1–40 deposits in preference to amyloid‐β 1–42 deposits [34]. However, with respect to AD, Zlokovic *et al*. have shown that clusterin functions at the blood–brain barrier to clear amyloid proteins [36] alongside ApoE [37]. It may bind other proteins due to its flexible structure [38], and is widely implicated as a biomarker for the diagnosis of AD [6, 7, 8, 10, 39]. Taking these together, raised plasma and CSF clusterin levels in patients may be indicative of tissue damage [40]. However, our observations support the view that clusterin is an indicator of early‐stage WM changes with multifactorial causes rather than specific for AD. It is also not unlikely that such accumulation of clusterin within vessel walls is due to its function as a chaperone protein during tissue remodelling and disease [2].

In addition to vascular amyloid β 1–40 in CAA, we found strong clusterin immunostaining within vessel walls of CADASIL subjects [12, 13]. We used high‐resolution confocal microscopy and EM to investigate the possibility that clusterin may be a component of GOM deposits. Our results indicate that despite strong immunostaining for both clusterin and N3ECD‐labelled GOM within the majority of vessel walls, these two antigens were not co‐localized within vessel walls. Our observations suggest the localization of vascular clusterin occurs as a distinct pathological process different from GOM formation [12]. It is possible that several disparate proteins would be identified by mass spectrometry of extracts of bulk samples of isolated cerebral blood vessels. We concur with the view that aggregated N3ECD proteins accumulate as GOM deposits, which then recruit other proteins in a ‘snowball effect’ so that at the end stage, there may be numerous proteins within GOM [12]. However, the aggregation of GOM may be limited at the vascular cell membranes as most deposits are between 0.2 and 2 μm in diameter [16].

Clusterin enrichment in blood vessel walls likely relates to its function as a chaperone to other proteins, which may be by‐products of ischaemic damage or waste proteins for clearance by the perivascular drainage pathway [17]. In addition, we observed intracellular expression of clusterin within vascular smooth muscle cells with some immunoreactivity within the nucleus. Cytoplasmic clusterin is thought to be protective against programmed cell death [1, 38], and nuclear clusterin is considered a pro‐cell death isoform [41]. Clusterin expression within the walls of arterioles appears common to many cerebral disorders, and therefore, while GOM may be juxtaposed to clusterin, our results suggest it is not a specific component of GOM.

In summary, we have shown high differential clusterin protein expression in some cerebrovascular disorders related to dementia. Although not emphasized previously, we particularly demonstrated that clusterin immunostaining was increased during WM damage. Our work does not support the notion that clusterin is a specific component within GOM deposits in CADASIL but it is found to be strongly associated with amyloid deposits in CAA. Our findings suggest that clusterin plays a role in different vascular‐based disorders, potentially in facilitating clearance of degraded or aggregated proteins. It is likely that genetic variation in the clusterin gene affects its ability to clear proteins at the blood brain barrier and via perivascular routes.

## Author contributions

The individual contributor statements are as follows:

LJLC: Analysis and interpretation of data, acquisition of the microvascular results and revising the manuscript at various stages of preparation.

JLT: Analysis and acquisition of the original IHC data in hereditary stroke disorders.

JYS: Sample preparation, technical assistance and interpretation of immunostaining.

AC: Analysis and additional interpretation of the data.

CH, GK, AB‐H and HK: Responsible for clinical and neuropathological analyses, provision of post‐mortem brain tissue and general advice on analysis.

VD: Assessment of vascular pathological scoring.

A E Oakley: Analysis/interpretation/acquisition of EM data and technical advice on imaging.

RNK: Drafting, revising the manuscript and interpretation of data, diagnosing the cases and obtaining funding.

## Disclosures

The co‐authors have no disclosures with regard to this report. The study was not industry sponsored. There are no conflicts of interest.

## Supporting information


**Figure S1.** Clusterin immunostaining in axons was assessed using a modified scoring scale according to the following scale: 0, no axonal staining (46 year‐old male cognitively normal young control); 1, infrequent, short punctate staining (81 year‐old male cognitively normal old control); 2, infrequent staining of individual axons (81 year‐old female small vessel disease dementia case); 3, patches of punctate staining with swollen axonal lengths (48 year‐old female Swedish hereditary multi‐infarct dementia); 4, tracts of densely stained and swollen axons but unstained areas still apparent (96 year‐old male with small vessel disease dementia); 5, complete areas of densely stained and swollen axons (59 year‐old female with pontine autosomal dominant arteriopathy microangiopathy and leukoencephalopathy).
**Figure S2.** Representative immunoblot of protein extracts from white matter. YC, young control; OC, old control; 95+, cognitively normal control aged >95 years; SVD, small vessel disease; CAD, CADASIL; Std, pooled sample of all cases used as a loading control. Anti‐clusterin antibody detected bands at 38 kDa in all cases, as well as a second band at 55 kDa in one CADASIL case. Following detection with clusterin, the membrane was stripped and re‐probed with anti‐α/β Tubulin was used as a loading control (antibody #2148S, Cell Signalling, Cell Signaling Technology, Inc., Danvers, MA, United States) which detected a 55 kDa band in all cases. Molecular weight marker is identified on the left with protein bands 100, 75, 50, 40, 35, 25 and 15 kDa.
**Figure S3.** Clusterin was found to stain around pial arteries suggesting clearance of the protein with bound proteins such as amyloid β 1–40 through the perivascular drainage route. (**A**) Clusterin (red) and smooth muscle alpha actin (green) immunostaining within pial vessels beneath the meninges of 77 year‐old female with cerebral amyloid angiopathy (CAA), Braak stage 6, CERAD frequent (DAPI counterstain). (**B**) Clusterin (red) and smooth muscle alpha actin (green) immunostaining within pial vessel beneath the meninges in CADASIL [61 year‐old male CADASIL case with R169C mutation (DAPI counterstain)]. (**C**) Clusterin (green) was found to accumulate around pial vessel walls of arterioles with amyloid β 1–40 (red), suggesting the bound proteins may be cleared by the perivascular drainage route, 88 year‐old male with cerebral amyloid angiopathy (CAA), Braak stage 6, CERAD frequent (no counterstain).
