# Supplementary figures and images for: Clusterin/Apolipoprotein J immunoreactivity is associated with white matter damage in cerebral small vessel diseases
Source: Neuropathol Appl Neurobiol. 2015 Jun 17;42(2):194–209. doi: 10.1111/nan.12248 (PMC4949672; doi:10.1111/nan.12248)

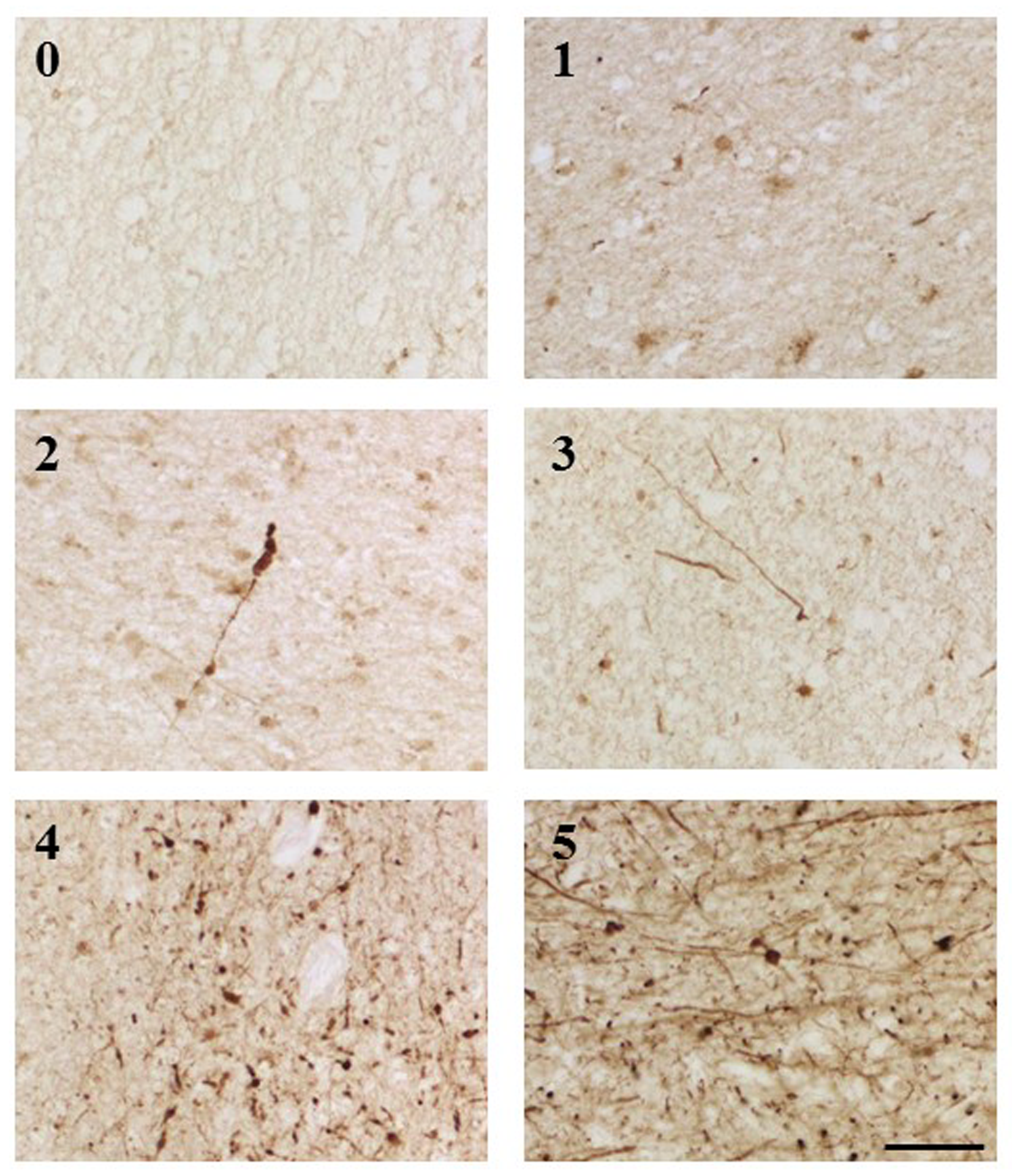

Supplement: Supplementary file 1 — Figure S1. Clusterin immunostaining in axons was assessed using a modified scoring scale according to the following scale: 0, no axonal staining (46 year‐old male cognitively normal young control); 1, infrequent, short punctate staining (81 year‐old male cognitively normal old control); 2, infrequent staining of individual axons (81 year‐old female small vessel disease dementia case); 3, patches of punctate staining with swollen axonal lengths (48 year‐old female Swedish hereditary multi‐infarct dementia); 4, tracts of densely stained and swollen axons but unstained areas still apparent (96 year‐old male with small vessel disease dementia); 5, complete areas of densely stained and swollen axons (59 year‐old female with pontine autosomal dominant arteriopathy microangiopathy and leukoencephalopathy). Figure S2. Representative immunoblot of protein extracts from white matter. YC, young control; OC, old control; 95+, cognitively normal control aged >95 years; SVD, small vessel disease; CAD, CADASIL; Std, pooled sample of all cases used as a loading control. Anti‐clusterin antibody detected bands at 38 kDa in all cases, as well as a second band at 55 kDa in one CADASIL case. Following detection with clusterin, the membrane was stripped and re‐probed with anti‐α/β Tubulin was used as a loading control (antibody #2148S, Cell Signalling, Cell Signaling Technology, Inc., Danvers, MA, United States) which detected a 55 kDa band in all cases. Molecular weight marker is identified on the left with protein bands 100, 75, 50, 40, 35, 25 and 15 kDa. Figure S3. Clusterin was found to stain around pial arteries suggesting clearance of the protein with bound proteins such as amyloid β 1–40 through the perivascular drainage route. (A) Clusterin (red) and smooth muscle alpha actin (green) immunostaining within pial vessels beneath the meninges of 77 year‐old female with cerebral amyloid angiopathy (CAA), Braak stage 6, CERAD frequent (DAPI counterstain). (B) Clusterin (red) and smooth [file NAN-42-194-s001.zip › NAN_12248-supp-0001-Supplementary Figure 1_ revised_Craggs L et al 2015.tif]

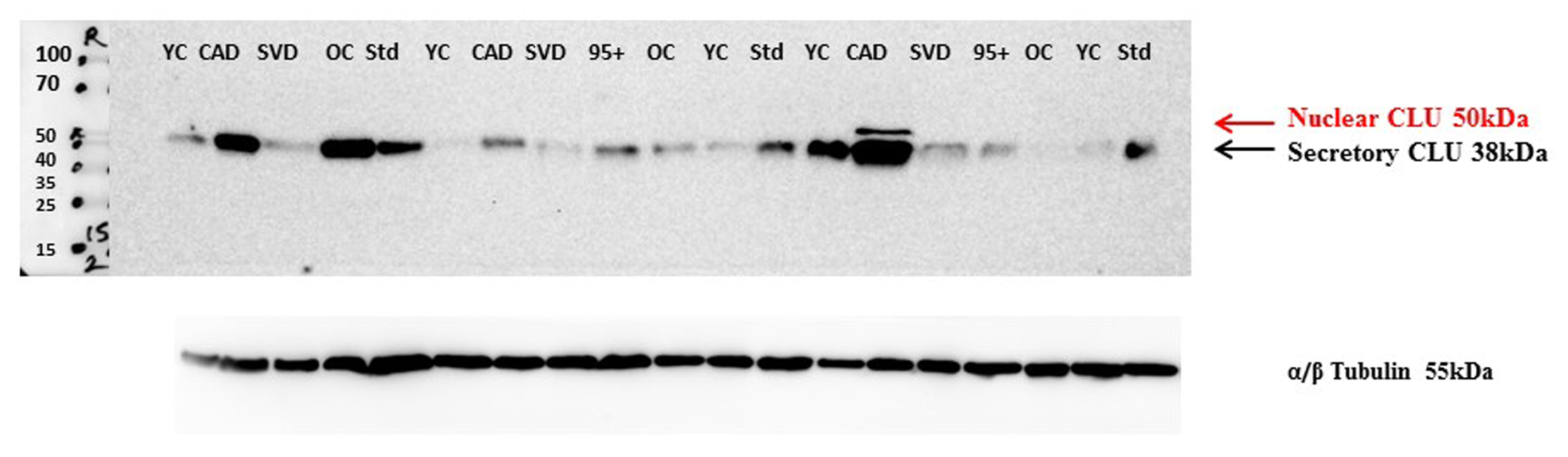

Supplement: Supplementary file 1 — Figure S1. Clusterin immunostaining in axons was assessed using a modified scoring scale according to the following scale: 0, no axonal staining (46 year‐old male cognitively normal young control); 1, infrequent, short punctate staining (81 year‐old male cognitively normal old control); 2, infrequent staining of individual axons (81 year‐old female small vessel disease dementia case); 3, patches of punctate staining with swollen axonal lengths (48 year‐old female Swedish hereditary multi‐infarct dementia); 4, tracts of densely stained and swollen axons but unstained areas still apparent (96 year‐old male with small vessel disease dementia); 5, complete areas of densely stained and swollen axons (59 year‐old female with pontine autosomal dominant arteriopathy microangiopathy and leukoencephalopathy). Figure S2. Representative immunoblot of protein extracts from white matter. YC, young control; OC, old control; 95+, cognitively normal control aged >95 years; SVD, small vessel disease; CAD, CADASIL; Std, pooled sample of all cases used as a loading control. Anti‐clusterin antibody detected bands at 38 kDa in all cases, as well as a second band at 55 kDa in one CADASIL case. Following detection with clusterin, the membrane was stripped and re‐probed with anti‐α/β Tubulin was used as a loading control (antibody #2148S, Cell Signalling, Cell Signaling Technology, Inc., Danvers, MA, United States) which detected a 55 kDa band in all cases. Molecular weight marker is identified on the left with protein bands 100, 75, 50, 40, 35, 25 and 15 kDa. Figure S3. Clusterin was found to stain around pial arteries suggesting clearance of the protein with bound proteins such as amyloid β 1–40 through the perivascular drainage route. (A) Clusterin (red) and smooth muscle alpha actin (green) immunostaining within pial vessels beneath the meninges of 77 year‐old female with cerebral amyloid angiopathy (CAA), Braak stage 6, CERAD frequent (DAPI counterstain). (B) Clusterin (red) and smooth [file NAN-42-194-s001.zip › NAN_12248-supp-0002-Supplementary Figure 2_ revised_Craggs L et al 2015.tif]

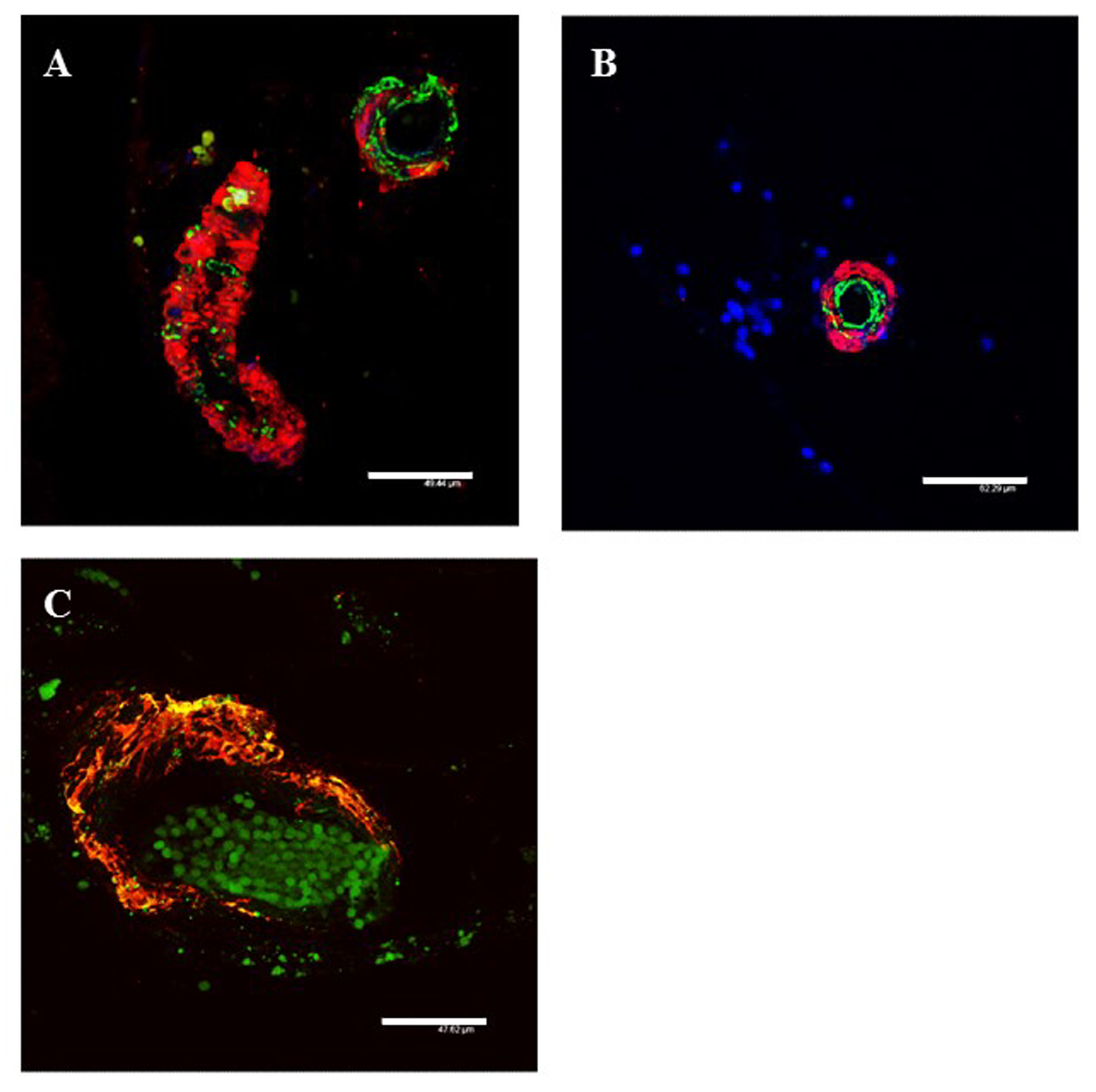

Supplement: Supplementary file 1 — Figure S1. Clusterin immunostaining in axons was assessed using a modified scoring scale according to the following scale: 0, no axonal staining (46 year‐old male cognitively normal young control); 1, infrequent, short punctate staining (81 year‐old male cognitively normal old control); 2, infrequent staining of individual axons (81 year‐old female small vessel disease dementia case); 3, patches of punctate staining with swollen axonal lengths (48 year‐old female Swedish hereditary multi‐infarct dementia); 4, tracts of densely stained and swollen axons but unstained areas still apparent (96 year‐old male with small vessel disease dementia); 5, complete areas of densely stained and swollen axons (59 year‐old female with pontine autosomal dominant arteriopathy microangiopathy and leukoencephalopathy). Figure S2. Representative immunoblot of protein extracts from white matter. YC, young control; OC, old control; 95+, cognitively normal control aged >95 years; SVD, small vessel disease; CAD, CADASIL; Std, pooled sample of all cases used as a loading control. Anti‐clusterin antibody detected bands at 38 kDa in all cases, as well as a second band at 55 kDa in one CADASIL case. Following detection with clusterin, the membrane was stripped and re‐probed with anti‐α/β Tubulin was used as a loading control (antibody #2148S, Cell Signalling, Cell Signaling Technology, Inc., Danvers, MA, United States) which detected a 55 kDa band in all cases. Molecular weight marker is identified on the left with protein bands 100, 75, 50, 40, 35, 25 and 15 kDa. Figure S3. Clusterin was found to stain around pial arteries suggesting clearance of the protein with bound proteins such as amyloid β 1–40 through the perivascular drainage route. (A) Clusterin (red) and smooth muscle alpha actin (green) immunostaining within pial vessels beneath the meninges of 77 year‐old female with cerebral amyloid angiopathy (CAA), Braak stage 6, CERAD frequent (DAPI counterstain). (B) Clusterin (red) and smooth [file NAN-42-194-s001.zip › NAN_12248-supp-0003-Supplementary Figure 3_ revised_Craggs L et al 2015.tif]
